# An Efficient Audio Coding Scheme for Quantitative and Qualitative Large Scale Acoustic Monitoring Using the Sensor Grid Approach

**DOI:** 10.3390/s17122758

**Published:** 2017-11-29

**Authors:** Félix Gontier, Mathieu Lagrange, Pierre Aumond, Arnaud Can, Catherine Lavandier

**Affiliations:** 1 LS2N, UMR 6004, École Centrale de Nantes, 44300 Nantes, France; felix.gontier@ls2n.fr; 2 LAE, AME, IFSTTAR, 44340 Bouguenais, France; pierre.aumond@ifsttar.fr (P.A.); arnaud.can@ifsttar.fr (A.C.); 3 ETIS, UMR 8051, Université Paris Seine, Université de Cergy-Pontoise, ENSEA, CNRS, 95000 Cergy-Pontoise, France; catherine.lavandier@u-cergy.fr

**Keywords:** acoustic monitoring, audio encoding

## Abstract

The spreading of urban areas and the growth of human population worldwide raise societal and environmental concerns. To better address these concerns, the monitoring of the acoustic environment in urban as well as rural or wilderness areas is an important matter. Building on the recent development of low cost hardware acoustic sensors, we propose in this paper to consider a sensor grid approach to tackle this issue. In this kind of approach, the crucial question is the nature of the data that are transmitted from the sensors to the processing and archival servers. To this end, we propose an efficient audio coding scheme based on third octave band spectral representation that allows: (1) the estimation of standard acoustic indicators; and (2) the recognition of acoustic events at state-of-the-art performance rate. The former is useful to provide quantitative information about the acoustic environment, while the latter is useful to gather qualitative information and build perceptually motivated indicators using for example the emergence of a given sound source. The coding scheme is also demonstrated to transmit spectrally encoded data that, reverted to the time domain using state-of-the-art techniques, are not intelligible, thus protecting the privacy of citizens.

## 1. Introduction

The advent of low cost acoustic sensors together with the need to better monitor and comprehend the acoustic environment of urban and wilderness areas give rise to the deployment of experimental sensor networks such as the SONYC (htttp://wp.nyu.edu/sonyc) [1] and CENSE (htttp://cense.ifsttar.fr) [2] projects. To do so, considering the so-called “sensor grid” approach [3,4] has several advantages. The sensor grid approach puts the focus on: (a) a distributed system of data acquisition with a large set of sensors; (b) the production and storage of a large dataset; and (c) its availability for intensive and open data analysis computation.

In such approach, the number of sensor nodes shall be extended as needed without having to change the hardware and software architecture, i.e., the approach shall be scalable. Second, the nodes shall be energy efficient, and, ideally, autonomous, to ease the deployment of the sensor grid to the desired topology. Third, the encoding scheme used to transmit the data from the sensor to the storage and processing servers shall be designed with care in order to guarantee the privacy of the citizens. At the same time, the data stored shall remain as rich as possible, while maintaining a low bitrate for efficient transmission and storage.

In the case of urban acoustic monitoring, the data of interest typically consist of standardized high-quality measurements of energetic indicators such as the equivalent sound level $L_{eq}$ in dB SPL or its A-weighted counterpart $L_{Aeq}$ in dBA. However, while these indicators have proven to correlate well with perceptual evaluations for negative impact sound environments [5], they are not sufficient to fully describe urban sound environments [6]. Many other variables can be derived to better account for previously implicit properties [7] including percentile values or time dynamics. Studies have been conducted to select relevant subsets of descriptors in sound environment characterization [8,9,10].

**Figure 1:**
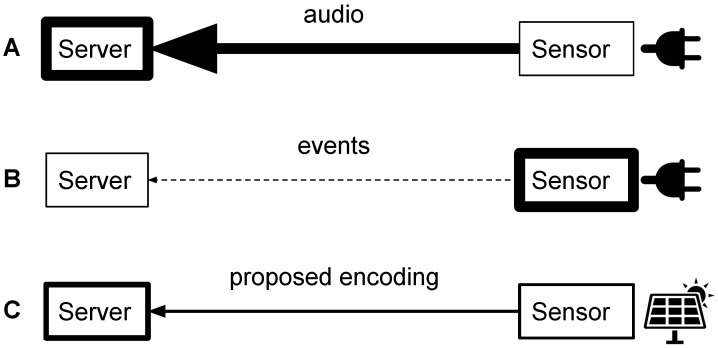
Three alternative implementations of the sensor grid approach for the monitoring of the acoustic environment: in (**A**) the raw audio is transmitted; in (**B**) only the detected events; and in (**C**) a compressed spectral representation. Thickness of arrows indicates bandwidth and thickness of boxes indicates the level of computation or storage required.

In addition, considering sound sources allows better assessing the human perception of the urban sound environment in terms of pleasantness and other perceptual indicators [11,12]. For instance, bird song and traffic noise with the same energy are perceived very differently. Most acoustic indicators used in current sound maps describe the global sound environment and lack information on its composition. The recognition and the estimation of the level of contribution of sources of interest is thus of great importance to fully model the studied acoustic environments (see for example [13,14,15]). Other indicators have been found to correlate with specific sound environment content [12] but do not achieve the level of performances of a well-designed source detection scheme in characterizing acoustic environments. This source detection step can be operated online (on the sensors) or offline (on the data servers), leading to different grid designs (Figure 1).

Performing the detection online (case B of Figure 1) is efficient in terms of data storage as only the detected events are transmitted. This approach is thus currently considered in several applications in the literature [1,16,17]. However, it requires the availability of decent computing resources on the sensors in order to perform the detection step which as of today’s efficiency of hardware does not allow them to be autonomous using power sources like solar panels. Furthermore, the detection is done once and cannot be recomputed during offline posterior analyses.

Performing the detection offline has several benefits. First, the sensor task is much simpler and can thus be implemented on low energy consumption processors. This means that the sensors can be autonomous in terms of energy, which greatly eases the deployment of the network. Second, it allows researchers to gather large amount of data that can be post-processed and studied further offline. Data can be re-analyzed following newer classification schemes or using new indicators. Though, transmitting the raw audio through the network (case A of Figure 1) has several disadvantages, in terms of required bandwidth, storage capabilities and privacy. Thus, for transmission from the sensors to the storage unit, the data shall be encoded in a more efficient way than lossy audio encoding standards [18]. Indeed, the audio signal is not mandatory for the computation of the acoustic indicators and the features needed for event recognition. In addition, as the data are transmitted using the network and stored, one must ensure that the intelligibility of potential speech utterances is lost during the coding process, in order to guarantee the privacy of the citizens.

The analysis of sources within a sound environment is generally linked with spectral content [19] and high temporal resolutions while acoustic indicators focus on more scarce but highly accurate measurements. We thus present in this paper an audio coding scheme specifically tailored for use in a sensor grid (case C of Figure 1). The proposed method computes and encodes third-octave bands $L_{eq}$ with controllable precision, enabling low bitrate transmissions while allowing both the derivation of other acoustic indicators and the implementation of offline audio event detection schemes. We report equivalent performance of several state-of-the-art classification models from features computed using raw audio data and encoded data at production level rates. Finally, according to a perceptual evaluation, the proposed coding scheme very strongly degrades the intelligibility, thus ensuring citizen privacy.

To promote reproducible research, the coder as well as the experiments needed to generate the figures is available to the research community (https://github.com/felixgontier/cense-coder). The remaining of the paper is organized as follows: Section 2 describes the proposed encoding scheme; the experimental protocol designed to validate the proposed approach is described in Section 3; and results are discussed in Section 4.

## 2. Proposed Encoding Scheme

### 2.1. Data Representation

The identification of sources from audio streams has been the subject of extensive research in the past for speech data [20], music data [21], and lately environmental data, in which the current work falls. Studied classification methods are diverse, ranging from time-dependant modeling with hidden Markov models [22] to “bag-of-frames” approaches [23,24]. Common architectures include learning-based classifiers such as support vector machines [25], Gaussian mixture models [26] or neural networks [27,28]. In addition, several type of features are considered to feed those classifiers. The most used are certainly spectral [29] or cepstral [30] representations of the signal. Among them, Mel spectrograms and their cepstral-domain derivation, the Mel-frequency cepstrum coefficients (MFCC), are the most widely used as of today. These representations effectively model the human cochlear response to sounds by grouping frequency components around critical bands in a logarithmic scale. Features can also be computed in other domains to better model signal properties. For instance, Cai et al. [31] adds spectral features related to harmonicity and salient frequencies, and [32] uses a matching pursuit (MP) algorithm to deduce time-domain features. Alternative data representations such as the scattering transform [33] show good results in environmental sound classification tasks [34]. Another promising solution is feature engineering via unsupervised learning, which Salamon et al. [35] implements with a clustering technique. More complex systems using multiple-domain data representation as well as unsupervised models have been found to handle unrestricted environments such as urban acoustic scenes efficiently [36]. A more detailed review of used methods is available in [37].

In the acoustic monitoring field, the measurement of energetic indicators is mandatory to the development of noise maps. The third-octave bands $L_{eq}$ appears suitable for this work’s purpose: in addition to being a relevant descriptor [38] for urban sound environment assessment, it allows for the computation of most cited indicators while representing reasonably small, fixed amounts of data to be transmitted. Interestingly, third-octave bands are closely related to MFCC as they both are logarithmic-scaled spectral representations of the audio signal and diverge only in their motivation, mathematical and perceptive respectively.

We therefore consider the calculation of spectral energetic indicators such as the 31 third-octave bands within the human audition range 20 Hz–20 kHz. “Fast” measurements of third-octave bands with an integration time of 125 ms are preferred to suit the temporal resolution needs of AED schemes, although “slow”, 1 s measurements are also considered to further study the effect on our application’s constraints.

### 2.2. Signal Analysis

**Figure 2:**
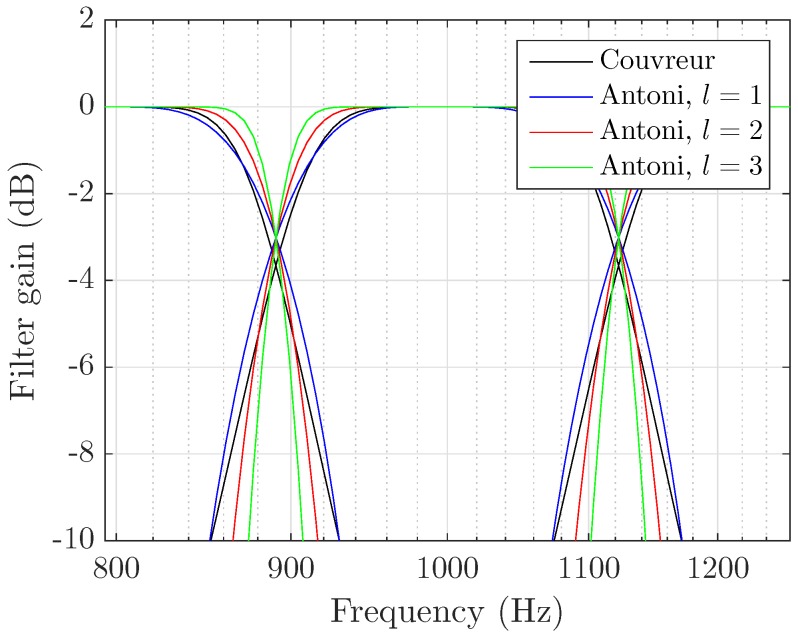
Comparison of Couvreur’s and Antoni’s implementations of third-octave filters. Frequency-weighting allows for arbitrary transfer functions and thus more accurate gains as standards impose.

A common way to implement third-octave analysis is to first design the highest octave three filters, and use them on progressively time-decimated versions of the input signal [39]. Antoni [40] presents an alternative analysis method based on direct frequency weighting, which yields a lower computational complexity as opposed to time filtering. The weights matrix design procedure complies with both ANSI S1.1-1986 [41] and IEC 61260-1:2014 [42] standards. It also meets the partition of unity principle over all frequency bins in the relevant range. Resulting filters for different parameter *l* values are compared with Couvreur’s implementation [43] with 2nd order Butterworth time-domain filters, as shown in Figure 2. The major difference is that gains at cutoff frequencies are fixed at the optimal −3 dB value by design.

The first step is to represent the signal in the frequency domain using a short-term Fourier transform (STFT). The continuously sampled audio is segmented into either 125 ms or 1 s frames to provide “fast” or “slow” acoustic measurements. The data are zero-padded to the next power of two to improve computational efficiency. The other parameters such as windowing functions and frame overlap are dependant on the specific constraints, and discussed in Section 3. The phase is discarded as it is not meaningful for third-octave analysis. Similarly, negative frequency components contain the same information as positive ones because the input signal is real. We then compute third-octave bands from the squared spectral magnitude by matrix multiplication with the frequency weights proposed in [40] for l=2 (when implementing said method, we found that squaring the $\phi_{l}\left(p\right),l\geq 1$ intermediate function (p. 887) was the correct approach to meet cutoff frequencies requirements, and we believe this was the author’s original intention). We include analysis for bands *i* from −17 to 13, i.e., center frequencies $f_{i}$ ranging from 20 Hz to 20 kHz with $f_{0}=1$ kHz. The representation is thus composed of 31 values.

### 2.3. Data Encoding

A Huffman coding scheme [44] is an efficient method to further reduce data rate while keeping the underlying information. At this point of the processing chain, the data take the form of a spectrogram with amplitude values represented on an arbitrary data type. It is therefore assimilable to a discrete distribution. By defining symbols as the possible values taken by the data, a discrete probability density function (PDF) can be expressed and estimated. The Shannon entropy then specifies the minimum average achievable output data rate for this given distribution, or average information content. The Huffman algorithm is an entropy coding scheme, as it uses the PDF to assign a code to each symbol so that the average output rate is the closest to the entropy. The resulting mapping function is called a Huffman dictionary.

The efficiency of the coding step thus directly depends on the entropy of the transmitted data. It is defined as $H=-\sum_{i}p_{i}{log}_{n}\left(p_{i}\right)$, where $p_{i}$ is the probability of appearance of a given symbol *i* and *n* is the numerical base in which the output code is represented. A lower entropy can be obtained with two factors. First, the dictionary size, i.e., the number of symbols must be kept low. This implies performing a quantization on the data resulting in a loss of precision. Second, the data distribution itself is important. Entropy decreases when very few symbols have a very high probability of appearance and is maximum for a uniform distribution.

**Figure 3:**
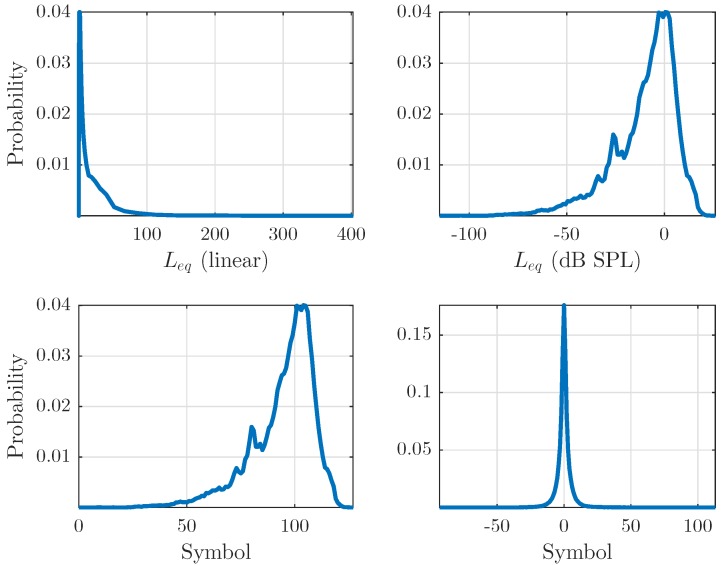
Example of estimated probability density functions of the data throughout the encoding step. Unchanged output of the representation step (**top**-**left**), concentrated towards very low values. PDF “flattening” effect induced by logarithm application (**top**-**right**). Here, values are mapped to the range $\left[0,2^{7}-1\right]\left(q=8\right)$ and rounded to perform quantization (**bottom**-**left**). Output of the Δ compression (**bottom**-**right**), with desirable probabilities as the input to a Huffman algorithm.

Considering an estimated PDF for our data, as shown in Figure 3 (top-left), immediately applying a linear quantization is clearly suboptimal, as the data mostly consist of very low values and a direct quantization results in most of the information being lost. We therefore flatten the PDF by taking the logarithm of the representation (top-right). This allows to better use the available range of symbols. A linear quantization is then applied with $2^{q-1}-1$ output values to obtain Figure 3 (bottom-left). Finally, we use a Δ-encoding algorithm along the time dimension to reduce redundancies between consecutive frames. This effectively concentrates higher probabilities on symbols around zero (Figure 3 bottom-right), yielding a higher amount of symbols at $2^{q}-1$ but nevertheless a smaller entropy. In the example shown here, the entropy before Δ-compression is $H_{log}=6.24\;sh$ which is reduced to $H_{\Delta }=3.54\;sh$.

**Figure 4:**
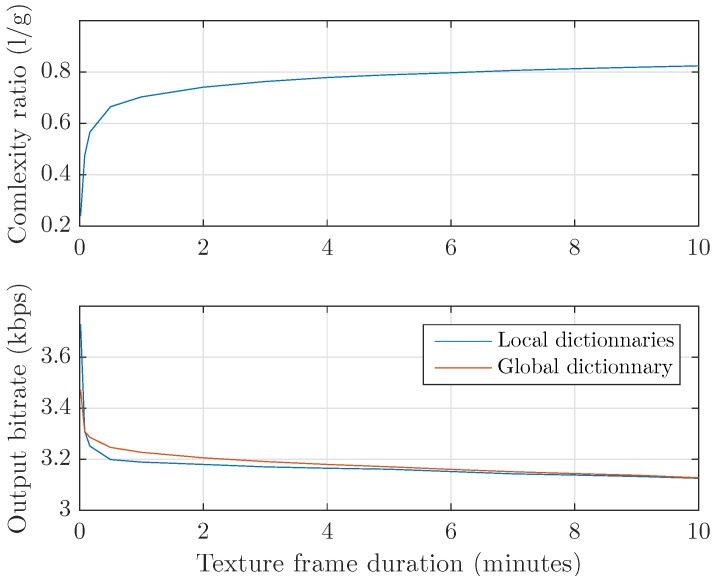
Performance comparison of locally and globally generated Huffman dictionaries. (**top**) The mean execution time ratio favors the use of frame-specific dictionaries for tested texture frame lengths. (**bottom**) The mean output bitrate is close for both algorithms, showing that the necessity of sending symbol-code pairs mostly compensates for their optimality.

Huffman encoding is then computed with either a frame-specific symbol-code dictionary or a fixed dictionary generated using a learning dataset. A comparison of both methods is shown in Figure 4. For most short texture frame durations, the local Huffman algorithm is found to be much faster. In fact, the encoding computational complexity is a function of the number of dictionary elements. A frame-specific dictionary only contains the symbols found in a given data packet, while a fixed dictionary must contain every possible symbol as it does not depend on the present data. The output bitrate is found to be close for both methods, as sending an optimal local dictionary balances the suboptimality of globally generated symbol-code pairs.

**Figure 5:**
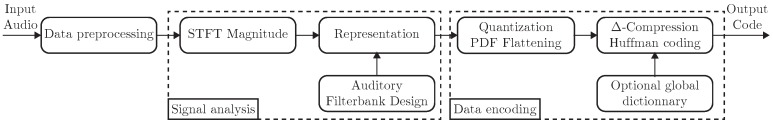
Overview of the encoding process.

The decoding process is quite straightforward, as both Huffman and Δ compressions are lossless and directly invertible. The entire coder scheme is summarized in Figure 5.

## 3. Validation Protocol

A set of metrics is computed to assess the efficiency of the proposed coder scheme and to determine the impact of the degrees of freedom given by the parameters of the proposed algorithm. Those parameters are as follow: (1) the desired word size prior to Huffman encoding, set by quantization for both signal representation choices; (2) the number of considered bands; and (3) the STFT parameters including frame duration, overlap and windowing function usage. This section details experimental protocols for the validation of bitrate, measurement error, audio event recognition and intelligibility developped to best suit the corresponding constraints.

### 3.1. Efficiency

The coder’s output bitrate as well as measurement error is computed for both data representations. This metric is composed of the spectral third-octave band analysis error and the additional loss yielded by quantization before the encoding process.

To assess the quality of the propoposed scheme’s spectral measurement precision, we compare it to a known reference: the Matlab *ita_toolbox* implementation [45]. This toolbox’s implementation shows less than 0.1 dB error on all bands when compared to a class 1 sound level meter software on 2 s third-octave bands measurements. We study both “fast” computation with 125 ms windows and “slow” 1 s analysis to assess the impact of time integration. Third-octave bands are computed using the proposed method as well as the *ita_toolbox* in the same experimental conditions, then averaged over time and compared to the toolbox’s measurements on longer 2 s extracts. The experiment is run for Gaussian white noise as well as urban environmental recordings: the former allows direct estimation bias evaluation, while the latter provides a simulation of real operating conditions. As the analysis frames are of short duration, poor frequency resolution and spectral leakage are expected when performing the STFT, particularly in the lower frequencies bands. For this reason, we evaluate the impact of windowing functions on each band measurement error in order to potentially mitigate this loss of precision.

To provide relevant statistics, these quantities are estimated with data from the 4500 audio recordings of the UrbanSound8k dataset [46] described in the next section and 4500 *iid.* Gaussian noise sequences.

### 3.2. Preserving Event Recognition Performance at Low Bitrate

To ensure that the proposed scheme allows the recognition of events using state-of-the-art methods, we consider the results achieved by several recognition systems on the UrbanSound8k dataset [46]. It features about 9 h of urban environmental sounds recordings separated in 8732 audio files ranging from 1 to 4 s each. The recordings are labeled in 10 classes (air conditioner, car horn, children playing, dog bark, drilling, engine idling, gun shot, jackhammer, siren, and street music), and distributed in 10 independent folds. A method and baseline results are also provided for the classification task. In this study, audio files are resampled at 44.1 kHz, normalized and only the left channel is considered for further analysis.

The aim of this experiment is two-fold: to evaluate the global loss of information induced by the coding process, and to evaluate the adequacy of third-octave bands indicators for classification. As studying the best performing classical models [47], deep learning approaches [27] or unsupervised feature learning is beyond the scope of this paper, we choose to replicate the baseline models and results proposed in [46].

**Figure 6:**
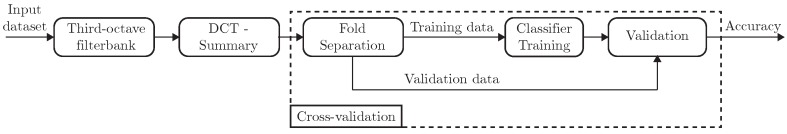
Classification process flowchart. Here third-octave bands are obtained by reversing the operations in Section 2.3 and cepstral coefficients are computed before training the model. Cross-validation is achieved by independantly training and testing the models with each of the 10 combinations of a 90–10% training–validation dataset separation.

The four classification models are as follow: a support vector machine with a radial basis function kernel, a random forest classifier with 500 trees, a decision tree and a k-nearest neighbors classifier with k=5. Values for the SVM parameter *C* and RBF kernel variance $\sigma^{2}$ are determined using a grid search. We optionally apply a discrete cosine transform to the critical band signal representation to obtain cepstra, known as Mel Frequency Cepstrum Coefficients or MFCC in the case of Mel spectrograms. The 25 first coefficients are kept, and summarized along time with several statistics such as the mean, variance, skewness, kurtosis, minimum, maximum, median, derivative mean and variance, second order derivative mean and variance operators. The feature vector is thus comprised of 275 values to reproduce available results and compare with the data produced by the proposed coding scheme. Models are trained for each setup using the 10-fold cross validation method provided by the authors of [46], that is, every combination of testing one fold on models trained with the other nine. A summary of the considered classification process is shown in Figure 6.

Mel spectrograms are also computed with the *rastamat* library [48] to assess the relative performance of third-octave bands. STFT analysis frames are obtained by applying a Hann window on 23.2 ms of signal with 50% (11.6 ms) overlap to allow for efficient phase recovery.

### 3.3. Inintelligibility

It is important that the proposed scheme ensures a high level of recognition of acoustic events but, as the data are transmitted over the network and potentially stored, the level of intelligibility of speech utterances potentially captured in the decoded stream should be as low as possible. Thus, intelligibility in decoded and reconstructed audio is also an important concern of this study.

The use of an audio recording as an evidence is considered by the forensic phonetics [49] field. The readers shall be aware that even recorded with a good quality, such recording remains a weak biometrical indicator [50]. However, even if the identity of the speaker cannot be asserted, one can attempt to transcribe the spoken utterances. Thus, we believe that it is important to demonstrate that the proposed encoding scheme do not allow such thing.

To decrease the intelligibility, several approaches can be considered. First, a speech versus non speech detector can be implemented on the sensor. If some speech is detected, the data are not transmitted. This approach is however prone to failure of the detector and will lead to unavailability of data during speech time periods, which might compromise the relevance of some indicators.

A second approach is to wisely choose the frame rate. Since a phoneme is about 100 ms to 200 ms long [51,52], a frame rate lower than 4 Hz should therefore dramatically reduce intelligibility.

Thus, the impact of the frame rate parameter of the proposed encoding scheme on intelligibility is evaluated. To do so, a perceptual test is conducted. For the evaluation, a dataset of clean speech utterances recorded in studio conditions is used. The dataset is composed of nine french sentences enunciated by three male and three female speakers. The sentences have a mean duration of 2 s and are composed of 5–12 words, averaging eight words. The 54 extracts are encoded using the proposed approach with four analysis frame rate parameters of, respectively, 50 Hz, 16 Hz, 8 Hz and 4 Hz leading to 216 audio excerpts being tested in total. However, as the recording conditions are the same for all extracts the speaker identity is considered as a source of variability rather than as a necessary factor in the test statistics. As a result, the objective is primarily to evaluate the nine sentences with four encoding frame rates, i.e., 36 parameters.

Several subjective intelligibility metrics are standardized in the literature such as the system proposed in [53]. However, metrics regarding the signal only (SIG) or background noise only (BAK) which are often used to evaluate speech enhancement schemes [54], are not suitable for this test due to the background and signal being mixed with an uncontrollable SNR by the encoding process. Given these conditions a subjective score on the whole signal is used similarly to the OVRL score in [53]. As decoding the transmitted audio induces a framing effect unevenly distorting the input utterance, a second metric is designed to assess the comprehension of words. For the perceptual test, 18 different sentences evenly distributed over the four frame rate parameters are selected for each subject. For each sentence, the participant first listens to the extract and is then asked to transcribe the utterance and to rate the perceived intelligibility from 1 (not intelligible) to 5 (intelligible). The first metric is computed as the ratio of correctly transcribed words and will be referred as the intelligibility ratio (IR) in this paper. The second metric is directly the score attributed by the listener, referred as the intelligibility score (IS).

Twelve subjects of age ranging from 17 to 60 which reported normal hearing participated to the listening test. The test was conducted using a Matlab interface displayed with a desktop computer and audio was played through *Beyerdynamics DT 770* headphones. The output level was set by the experimenter at the same level for all subjects. The mean duration of the test was about 8 min.

**Figure 7:**
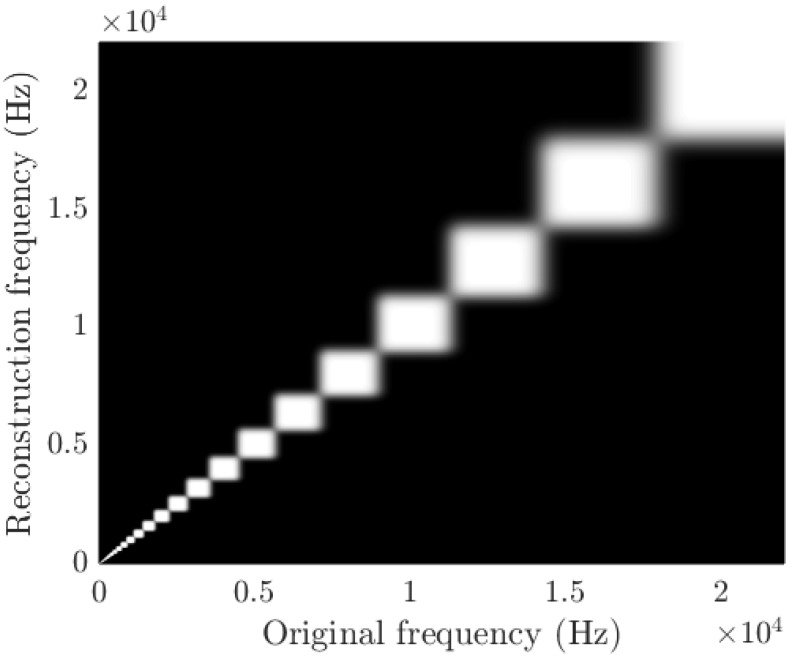
Third-octave bands analysis and approximate inverse transformation effects on energy location. Here, a brighter gray indicates that part of the energy contained at the corresponding frequency of the original signal is transferred to the reconstructed signal at the indicated frequency. This process yields an important and heterogeneous loss in resolution, particularly at higher frequency points.

This test requires an implementation of the inverse operation of the coding process to provide the listener with an estimate of the time-domain signal. To do so, a linearly-scaled spectrogram from the band-focused representations is estimated. This estimation is achieved by multiplying the spectral representation by the scaled transpose of the forward transformation matrix. A loss of resolution increasing with frequency is induced due to the shape of the transformation, as illustrated by Figure 7. Each white square can be interpreted as the filter applied to obtain a single third-octave band. The filtering process will average the energy content of the signal over the corresponding band. As a result the energy located at a specific frequency is impossible to recover and the best inverse approximation is to split the third-octave band’s observed energy into all the corresponding frequency bins with the same proportions as during analysis. This leads to the phenomenon seen in Figure 7 where part of the energy of a frequency bin in the original signal is found in all other bins of the same band at reconstruction, in almost equal proportions. The signal phase is recovered with the Griffin–Lim algorithm [55] if analysis overlap is used and white noise spectrogram scaling if not. The time-domain signal is then retrieved using the overlap-add method. When using overlap to compute third-octave bands, framing effects produced by rectangular windowing are reduced by convoluting the signal with a Hann windowing function prior to inverse-STFT computation.

## 4. Results

### 4.1. Coder Efficiency

**Figure 8:**
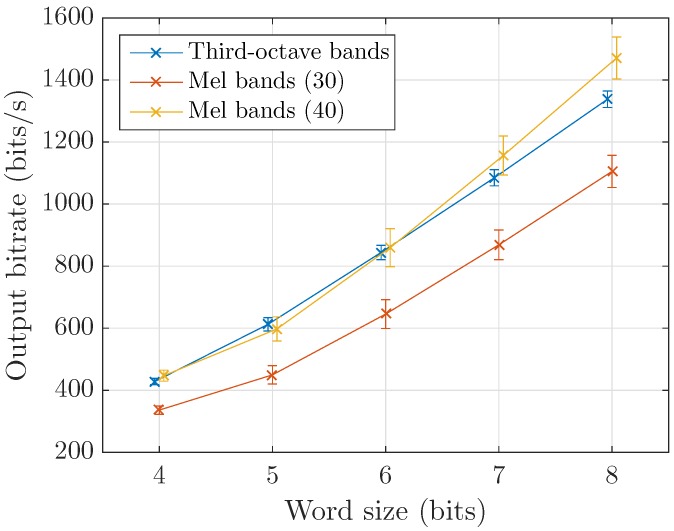
Coder output bitrate as a function of quantization for third-octave and Mel bands with 8 frames per second.

The main indicator of performance is the bitrate obtained at the output of the coder. The three varying factors studied are the data word size *q*, the number of bands and the effect of reducing time-resolution by averaging analysis frames. Figure 8 shows estimations of the output bitrate for different values of *q*. To match third-octave bands computation principle where a 125 ms (fast) or 1 s (slow) analysis is standardized, Mel frames are averaged over time. Because we use the most common parameters, namely 23.2 ms window with 50% overlap, the closest achievable rate considering simple averaging is 7.74 frames per second. Third-octave representation on 31 bands yields an overall higher size than their Mel equivalent, here estimated for 30 bands. It however compares with the 40-Mel bands representation which is the most used in sound event recognition literature. This higher bitrate is likely due to the distribution observed by the data prior to Huffman encoding.

A second set of parameters influences directly the time-frequency resolution of the analysis. By choosing a frame rate and number of bands, one can effectively control the size of the periodically transmitted data. We evaluate the bitrate for 10 to 40 Mel bands and a frame rate from 2 to 10 per second, with fixed q=8. As can be expected, the bitrate for a given word size *q* can be approximated as a linear function of the representation dimensions for one second of analysis. Small variations are induced by data distributions on a per-frame level and their impact on the Huffman algorithm.

This experiment aims at an initial assessment of potential tradeoffs between bitrate and preservation of valuable information which will be further discussed in Section 4.2.

**Figure 9:**
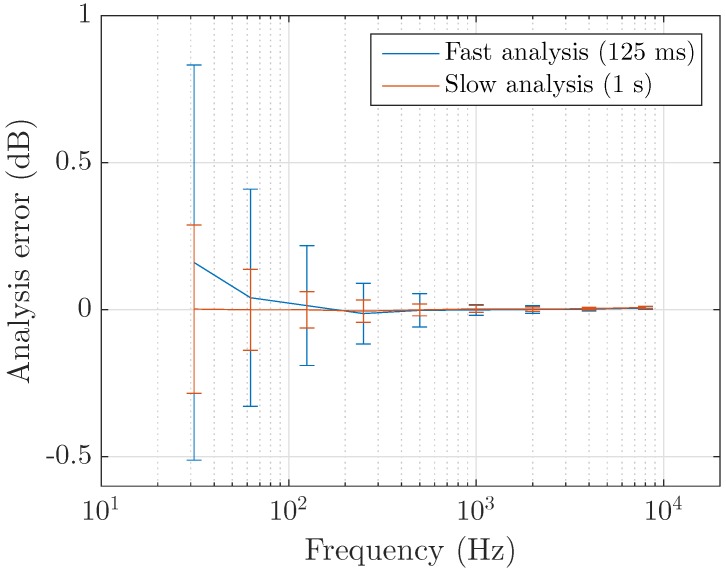
Measurement error of third-octave bands over two-seconds white noise extracts, with rectangular window and no overlap. The analysis of short frames has an effect on energy estimation at low frequencies that is of lower importance as the frequency resolution increases.

**Figure 10:**
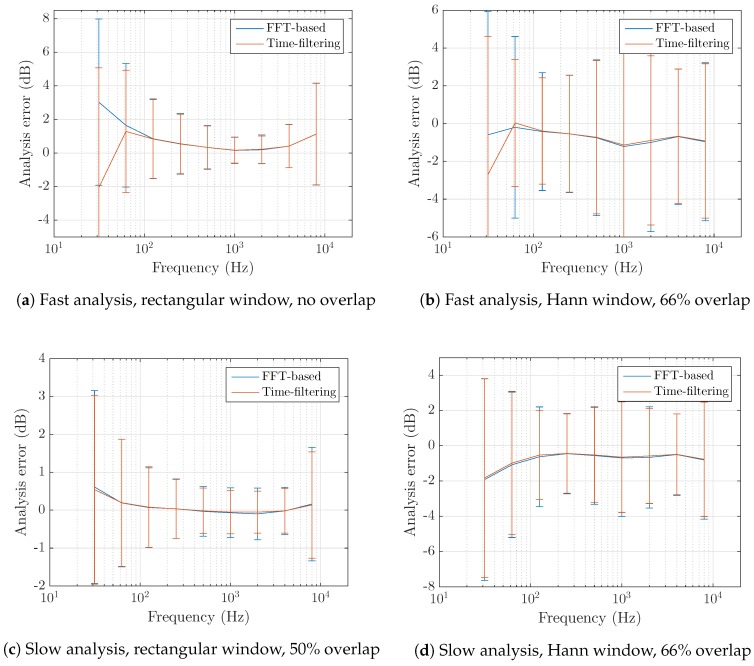
Measurement error of third-octave bands over two-seconds environmental sounds recordings (US8k dataset). For most settings, the FFT-based method yields similar errors compared to the time-domain filtering reference.

The measurement error is now computed for third-octave bands. Results are displayed in Figure 9 for white noise extracts and in Figure 10 for environmental sounds. Only full-octave bands are shown for the sake of visibility. When computing the slow analysis, we find that applying overlap attenuates the error. This is not observed for fast analysis where the error is similar or higher with the use of overlapping frames.

When performing a short time analysis, strong errors appear in low frequency bands where the precision of the DFT is poor. The slow analysis yields better estimations as it provides a globally better resolution and thus also reduces the impact of spectral leakage effects in this region. When considering white noise the error is low on the whole frequency range. Its mean over the testing samples is close to zero and its standard deviation increases in low frequencies, confirming the above-mentioned considerations. This effectiveness does not translate well to environmental sound analysis, most likely due to the sound level disparities absent in the former case but omnipresent in the latter. Spectral leakage induces a correlation between close frequency components. In lower frequencies, large differences between adjacent frequency bins can therefore cause important errors on the third-octave bands computation. This issue is however not specific to Fourier transform-based schemes as it is also encountered with a time domain filtering approach.

To mitigate this phenomenon, the choice of the window function shall be studied further. For a fast analysis with no overlap, the rectangular window seems a reasonable choice at first because of its energy conservation qualities. While it yields the lowest error in high frequency bands, its important spectral leakage effect makes it unprecise in lower frequency regions. Non-flat windows better account for this issue but require assuming that the signal is stationary in a given frame. The use of overlap then results in better estimates at the cost of an increased data size. For slow measurements, considering the rectangular window is less harmful to band analysis due to an increased frequency resolution.

The comparison of the error achieved by the proposed scheme to that of third-octave bands estimated by a reference time-filtering algorithm is now considered. It results in important similarities. For fast measurements and a rectangular window (Figure 10a), a student *t*-test (p<0.05) shows that the errors are not significantly different between 125 Hz and 12 kHz bands. For the lower frequency bands, the two methods yield different error distributions but of similar importance. Using a Hann window function results in lower error in the low frequency range but adds a bias to the previously accurate band measurements. Again comparing the two computation methods shows a significant difference in the bands below 100 Hz only. A slow analysis results in statistically equivalent errors between the two methods apart from the very low frequencies: p=0.011 with the rectangular window (Figure 10c), p=0.003 at 20 Hz and p=0.021 at 25 Hz for the Hann window and 66% overlap (Figure 10d).

The additional error caused by the encoding steps after obtaining the desired data representation is now studied. The only lossy operation applied is quantization, which is defined by an output word size *q* in bits. However, it corresponds to the word size *after* Δ-compression, meaning that the data are in fact quantized on $2^{q-1}$ values. Since these measurements are already expressed in dB, the error ε is computed as:

$$\varepsilon =|x_{q}-x|$$

where $x_{q}$ and *x* are the quantized and clean representations repectively. $x_{q}$ is given by:

$$x_{q}\left(n\right)=\frac{\Delta_{x}}{2^{q-1}-1}\mathrm{round}\left(\frac{\left(2^{q-1}-1\right)x\left(n\right)}{\Delta_{x}}\right),x\in \left[0,\Delta_{x}\right]$$

**Figure 11:**
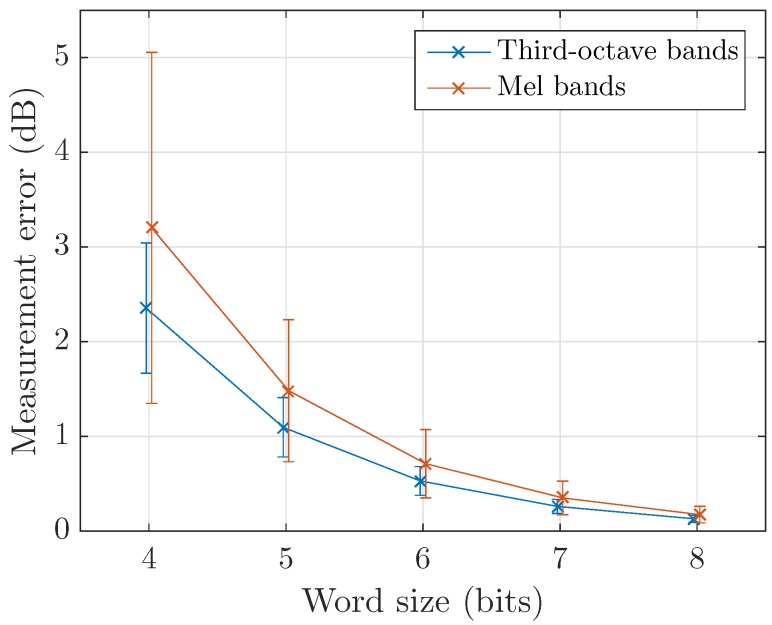
Measurement error induced by encoding for different quantization resolutions.

Figure 11 shows an estimation of ε as a function of *q* for third-octave and Mel bands. In both cases, the mean and standard deviation seem to decrease by a factor of 2 as *q* increases. To better understand this phenomenon, let us model *x* as a uniform distribution such as $x∼U\{0,\Delta_{x}\}$. While this is not exact, it matches our objective when using a logarithm to flatten given PDFs. The error ε will follow a uniform distribution $U∼\{0,\frac{\Delta_{x}}{2\times (2^{q-1}-1)}\}$. Therefore, the mean and standard deviation are:

$$\left\{\begin{array}{c} \mu_{\epsilon }=\frac{\Delta_{x}}{4\times (2^{q-1}-1)}\\ \sigma_{\epsilon }=\frac{1}{12}\frac{\Delta_{x}}{2\times (2^{q-1}-1)} \end{array}\right.$$

justifying the decaying ratio as *q* increases. In practice, we assume that $\varepsilon =f(\Delta_{x},\frac{1}{2^{q-1}-1})$ with the heterogeneity of the data’s PDF inducing small variations.

As $\Delta_{x}$ is sampled for each analysis frame in our implementation, the error has the same statistics across all bands. It is to be added to the measurement error discussed in the same section before comparing to the values specified in the IEC 61672-1:2013 [56] standard on class 1 and 2 sound level meters.

### 4.2. Event Recognition

The same set of parameters as in bitrate evaluation is used to study the impact of their tuning on sound event recognition performance. This experiment aims at finding ways to further reduce the encoded data size without strongly affecting detection performance. Results of the four presented classification methods (Section 3.2) are provided for the sake of completeness.

**Figure 12:**
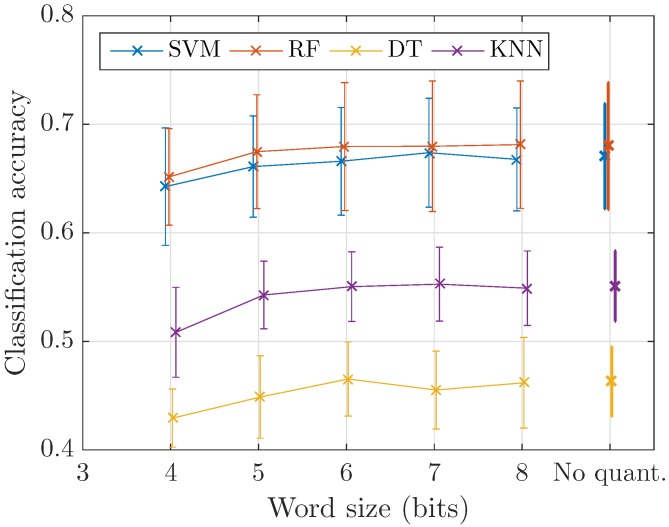
Classification accuracy as a function of word size before encoding. The baseline plotted on the right is computed without quantizing the representation.

First, we observe the impact of quantization on classification accuracy. The models are trained on the most complete implemented representation to replicate the results of [46], i.e., 40 Mel bands, 23.2 ms, 50% overlap analysis and no time averaging (85 frames per second). Figure 12 shows that equivalent accuracies are obtained for all considered values of the quantization parameter *q* down to 5 bits. The effect of changing the time-frequency resolution of the representation is presented in Table 1. In this table, equivalence of performance with respect to the best performing setting (in red) is depicted with a bold font evaluated using the following procedure. The null hypothesis that the subtraction of the two compared distribution (the distribution of the setting we consider minus the distribution of the best performing one) comes from a normal distribution with mean equal to zero and unknown variance is evaluated using the paired-sample *t*-test at the 0.05 significance level. If the null hypothesis is not rejected, the setting is considered as equivalent in terms of performance to the best performing one.

Similar to baseline results, the random forest and SVM classifiers are the best performing systems at 0.69±0.06 and 0.68±0.04 respectively. However, we find that given our setup, reducing the number of analysis bands up to a given level often does not induce a severe drop of performance. The accuracy of the decision tree classifier is a good example of this behavior, with best performances for 10 Mel bands only and a 4% lower accuracy for 40. Other models trained with 20 to 40 Mel bands as a representation show no significant performance difference. Time decimation can also be considered, as the original representation can be averaged and consistently yield a good classification accuracy. The loss of information can be considered negligible for frame-per-second values as low as 20 Hz or 10 Hz depending on the method. Even below, the classification accuracy only drops by one to a few percents. This means that we can effectively divide the data size by a factor of at most 10 without strongly affecting sound event recognition performance. It also provides us with a preliminary confirmation of the possibility for “fast”, 8 Hz third-octave bands cepstra to match MFCC performances.

### 4.3. Third-Octave Bands as Base Descriptors

**Figure 13:**
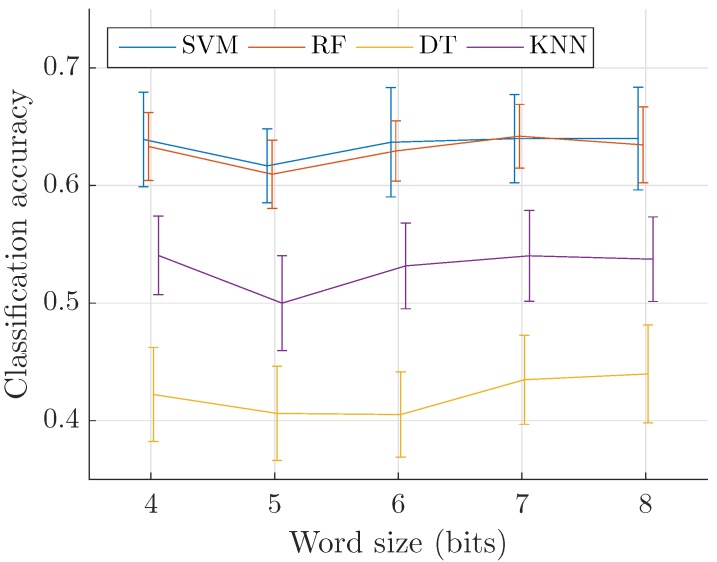
Classification accuracy with third-octave bands and varying word size.

We now compare the efficiency of third-octave bands at differenciating sound sources in urban acoustic environments to that of Mel spectrograms. Following the previous discussions, the classification task is run on corresponding cepstrum coefficients, computed with the following parameters: 125 ms analysis frames with rectangular windowing and no overlap are used to perform the STFT, and 31 third-octave bands (20 Hz–20 kHz) are considered. Figure 13 displays similar results to the 40 bands, 8 fps Mel spectrograms seen in Table 1 for all classification schemes.

To further analyze both representations advantages, the confusion matrix is a reliable tool. It aims at providing information regarding class-by-class accuracy and misclassification rates. We thus compute these metrics for the SVM classifier, with predictions accumulated over the ten folds in test configuration. The analogous designs of the two descriptors is highlighted by close one-versus-one differentiation performances, with a slightly lower accuracy for third-octave bands on average. Both representations yield best results for the *Gun shot* class with 88.2% for third-octave cepstra and 86.6% for Mel cepstra. Their poorest accuracy is on the *Air conditioning* class with 32.0% and 38.1% respectively. However, a noticeable difference between them is that on the log-frequency scale the bandwidth of Mel filters narrows as frequency increases, while third-octave are evenly distributed. The effect of this can be seen between classes *Drilling* and *Jackhammer*. These sounds involve important low-frequency information which can generally differentiate them, leading third-octave descriptors to perform better. Conversely, using Mel-based cepstra improves globally *Air conditioning* recognition as most of its defining components are situated in higher frequencies.

Both descriptor are thus found to have similar representational capabilities despite minor differences due to their respective designs.

### 4.4. Inintelligibility

The perceptual test (decoded speech utterances for various conditions can be listened to at https://felixgontier.github.io/cense-coder/) then evaluates the impact of analysis frame rate on intelligibility using the average intelligibility score (AIS) given by the subjects and the intelligibility ratio (IR) computed as the number of correctly transcribed words versus the number of words in the spoken utterance. Given the test conditions presented in Section 3.3 and the number of subjects, the mean and standard deviation are computed on an average of 54 results per frame rate parameter: 12 subjects evaluating each analysis resolution 4.5 times on average.

**Figure 14:**
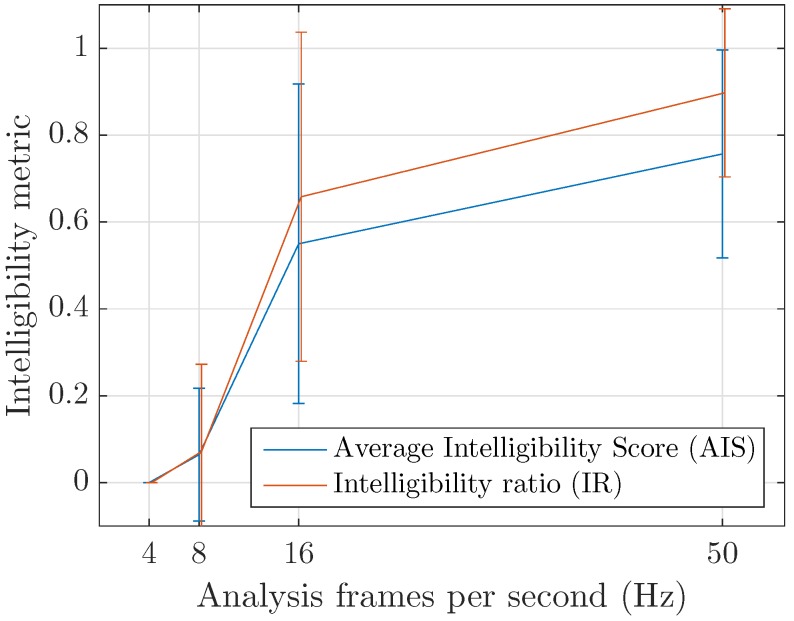
Mean and standard deviation of the Normalized Average intellibility score (AIS) given by the subjects and the inteligibility ratio (IR) as a function of the frame rate. Inintelligibility appears at around 10 Hz, corresponding to the average duration of a phoneme.

The results of the perceptual test, visible in Figure 14, confirm the qualitative evaluation of speech properties discussed in Section 3.3. Phonemes separation is indeed mandatory in order to have a good intelligibility of speech. This implies an analysis frame rate significantly larger than the phonemes rate the considered language. A limit at about 10 Hz corresponding to a frame duration of 100 ms is observed, as the studied 8 Hz setting is found to be almost completely inintelligible as very few words are understood correctly at this rate. We assume that the correctly identified words are a result of coincidentally adequate frame timings, i.e., analysis frames matching the precise location of phoneme utterances for a short duration. Similarly, framing effects induced by the proposed processing scheme yielded errors for higher frame rates transcriptions and a globally lower perceptual intelligibility.

## 5. Conclusions

An audio encoding scheme has been described and motivated for use in a sensor grid approach to tackle the problem of large scale acoustic monitoring. It has been designed to transfer data from acoustic sensors to processing and storage servers in an efficient and effective manner. It relies on a lossy audio coding scheme designed to preserve the privacy of the citizens while retaining the needed amount of acoustic precision to compute standard acoustic descriptors and to allow the recognition of events of interest at a state-of-the-art level of recognition accuracy.

It is based on a third octave spectral representation computed at an adjustable frame rate, encoded using an Huffman encoding scheme after logarithmic compression and differential encoding.

The proposed approach has the following benefits:

- Precision: The impact of the quantization step for 8 bits codewords is found to be very small (<0.2 dB), and thus:
  - does not impact the quality of the acoustics indicators (Section 3.1 and Section 4.1); and
  - enables sound event recognition with the same quality than directly from the spectrogram (Section 3.2, Section 4.2 and Section 4.3).
- Efficiency: Using the high precision parameters, the bitrate is about 1.4 kbps and 0.4 kbps for fast and slow modes respectively (Section 4.1).
- Privacy: At all operating modes, the reverted audio signal is found to be inintelligible according to a perceputal test done using clean speech recorded at very low background level. Additionaly, the sensors being typically hanged at 4 m high in a usually very noisy environment, we believe that the intelligibility risk of the proposed approach is negligible (Section 3.3 and Section 4.4).

The encoding scheme is designed to be easy to implement and to lead to an efficient data format for storage and sharing across the research community. To encourage its use for other research projects, an open source Matlab and Python implementations of the coder are available.

This scheme will be implemented with a large-scale sensor grid as part of the CENSE project, where sensors are designed to be low-cost and energy efficient. The aim of the project is to create noise maps by linking standard acoustic measurements with novel perceptually motivated metrics. The presented coding scheme will enable a thorough study of the correlation between sound sources and context with pleasantness as perceived by the citizens to better understand the content of urban acoustic scenes and its perception by city dwellers.

To do so, future work will focus on the use of such data encoder in a sensor grid approach as part of the CENSE project where its effectiveness will be assessed in field conditions. As far as the proposed encoding scheme is concerned, the usefulness of more sophisticated encoding paradigms should also be studied in order to further improve bitrate efficiency, as well as the effect of multiple channel coding and its interest in sensor grids applications.

## Figures and Tables

**Table 1:** Classification accuracy in percentage for different models: SVMs (**a**); Random Forests (**b**); Decision Trees (**c**); and Nearest Neigbors (**d**) with respect to varying representation resolutions. Numbers in red indicate best performance and numbers in bold indicate that no statistically significant difference is found when comparing to the best performing setting (p<0.05).

(**a**)								
SVM	Frames per second							
		2	4 (4.1)	6 (6.1)	8 (7.7)	10 (9.5)	20 (21)	85
Mel bands	10	55 ± 3	60 ± 3	61 ± 4	62 ± 3	62 ± 4	**63 ± 6**	**65 ± 6**
	20	58 ± 4	62 ± 4	63 ± 4	64 ± 4	63 ± 4	**65 ± 5**	**67 ± 6**
	30	60 ± 3	64 ± 4	64 ± 4	**65 ± 4**	**65 ± 3**	**67 ± 4**	**68 ± 4**
	40	60 ± 3	63 ± 4	**64 ± 4**	64 ± 4	**64 ± 4**	**66 ± 4**	**68 ± 5**
(**b**)								
RF-500	Frames per second							
		2	4 (4.1)	6 (6.1)	8 (7.7)	10 (9.5)	20 (21)	85
Mel bands	10	60 ± 3	62 ± 3	62 ± 3	63 ± 3	63 ± 3	**65 ± 4**	**67 ± 5**
	20	61 ± 4	63 ± 3	64 ± 3	64 ± 3	64 ± 4	**66 ± 6**	**69 ± 6**
	30	62 ± 3	63 ± 3	64 ± 3	64 ± 3	64 ± 4	**67 ± 5**	**69 ± 6**
	40	62 ± 4	63 ± 4	63 ± 4	64 ± 4	63 ± 4	**67 ± 6**	**68 ± 6**
(**c**)								
DT	Frames per second							
		2	4 (4.1)	6 (6.1)	8 (7.7)	10 (9.5)	20 (21)	85
Mel bands	10	42 ± 3	**46 ± 4**	46 ± 2	44 ± 3	45 ± 3	**46 ± 5**	**49 ± 3**
	20	43 ± 5	43 ± 3	43 ± 2	44 ± 3	45 ± 3	45 ± 5	**47 ± 5**
	30	42 ± 4	43 ± 2	44 ± 3	45 ± 5	43 ± 3	43 ± 4	**45 ± 5**
	40	42 ± 6	43 ± 3	43 ± 3	42 ± 3	44 ± 3	46 ± 3	**46 ± 4**
(**d**)								
KNN-5	Frames per second							
		2	4 (4.1)	6 (6.1)	8 (7.7)	10 (9.5)	20 (21)	85
Mel bands	10	43 ± 2	51 ± 4	53 ± 4	53 ± 5	53 ± 4	54 ± 4	**56 ± 3**
	20	44 ± 3	52 ± 4	53 ± 3	54 ± 4	**54 ± 4**	**55 ± 4**	**58 ± 4**
	30	45 ± 4	54 ± 5	**55 ± 5**	**55 ± 4**	**55 ± 4**	**56 ± 4**	**56 ± 4**
	40	46 ± 3	53 ± 5	**55 ± 5**	**55 ± 4**	**55 ± 5**	**57 ± 4**	**57 ± 3**
